# Perioperative Exhaled Nitric Oxide as an Indicator for Postoperative Pneumonia in Surgical Lung Cancer Patients: A Prospective Cohort Study Based on 183 Cases

**DOI:** 10.1155/2022/9149385

**Published:** 2022-09-04

**Authors:** Gui-Xian Liu, Yue Yang, Lei Chen, Mi-Qi Gu, Jin-Tao He, Xin Wang

**Affiliations:** ^1^Southwest Medical University, Luzhou 646000, Sichuan, China; ^2^Sichuan Cancer Hospital, Chengdu 610041, Sichuan, China; ^3^Department of Thoracic Surgery, Sichuan Cancer Hospital, Chengdu 610041, Sichuan, China

## Abstract

**Introduction:**

This study is conducted to investigate the correlation between perioperative fractional exhaled nitric oxide and postoperative pneumonia (POP) and the feasibility of perioperative FeNO for predicting POP in surgical lung cancer patients.

**Methods:**

Patients who were diagnosed with non-small-cell lung cancer (NSCLC) were prospectively analyzed, and the relationship between perioperative FeNO and POP was evaluated based on patients' basic characteristics and clinical data in the hospital.

**Results:**

There were 218 patients enrolled in this study. Finally, 183 patients were involved in the study, with 19 of them in the POP group and 164 in the non-POP group. The POP group had significantly higher postoperative FeNO (median: 30.0 vs. 19.0 ppb, *P* < 0.001) as well as change in FeNO (median: 10.0 vs. 0.0 ppb, *P* < 0.001) before and after the surgery. For predicting POP based on the receiver operating characteristic (ROC) curve, a cutoff value of 25 ppb for postoperative FeNO (Youden's index: 0.515, sensitivity: 78.9%, and specificity: 72.6%) and 4 ppb for change in FeNO (Youden's index: 0.610, sensitivity: 84.2%, specificity: 76.8%) were selected. Furthermore, according to the bivariate regression analysis, FEV1/FVC (OR = 0.948, 95% CI: 0.899–0.999, *P*=0.048), POD1 FeNO (OR = 1.048, 95% CI: 1.019–1.077, *P*=0.001), and change in FeNO (OR = 1.087, 95% CI: 1.044–1.132, *P* < 0.001) were significantly associated with occurrence of POP.

**Conclusions:**

This prospective study revealed that a high postoperative FeNO (>25 ppb), as well as an increased change in FeNO (>4 ppb), may have the potential in detecting the occurrence of POP in surgical lung cancer patients.

## 1. Introduction

Lung cancer (LC), a leading cause of cancer-related deaths worldwide, has been one of the most health-threatening and death-causing diseases to humans with the highest ranking of morbidity and mortality rates in China and unbearable social and economic burden globally [1, 2]. For the management of this disease, surgery is still the cornerstone for LC patients, especially for those with early-stage cancer, which is curable and resectable. Postoperative pneumonia (POP) is one of the most concerned postoperative complications for thoracic surgeons. As the consequences of POP, patients would suffer from increased mortality risk, hospitalization expenses, and prolonged length of hospital stay [3]. Establishing a model to predict which patients are at high risk for POP may contribute to making plans to reduce risk and allocating resources for postoperative care [4, 5].

Exhaled nitric oxide (NO) is produced from the lungs including bronchial epithelium, vascular endothelium, and pulmonary immune cells. It plays a crucial role in regulating pulmonary and bronchial smooth muscle and modulation of inflammation through alteration of leukocyte adhesion [6]. The expression of NO was at a relatively low output and could be induced by kinds of mediators via different NO pathways including NO production and consumption [7].

In clinical settings, fractional exhaled nitric oxide (FeNO) is widely used as a complementary tool in the diagnosis and monitoring of eosinophilic inflammation and in determining the response to steroid therapy for patients suffering from asthma [8]. Except for the application in asthma, a number of studies [9, 10] have discussed that FeNO may act as a marker of lung inflammation and injury. In recent years [11], molecules of exhaled breath have been studied for the management of other clinical settings, for example, the surgical patients. The measurement of these molecules is convenient and easy-to-use and might provide us a new method for the better perioperative management of surgical LC patients [11].

As surgical procedures, anesthesia and postoperative pulmonary complications may influence the production and consumption of FeNO. Thus, we conducted this prospective research to determine the relationship between FeNO and POP and investigate the application of this variable for predicting the occurrence of POP in surgical LC patients via measuring NO from exhaled breath of patients before and 24 hours after the surgery.

## 2. Patients and Methods

### 2.1. Ethical Review

This single-center prospective cohort study was approved by the institution's Clinical Trials and Biomedical Ethics Committee (No. (2017-403)). The authors declared that the research adheres to the tenets of the Declaration of Helsinki, with written informed consent obtained from the patients.

### 2.2. Patients

Each subject should meet all the inclusion criteria and none of the exclusion criteria for this study. Under no circumstances can there be exceptions to this rule. Inclusive criteria were listed as follows: (1) diagnosed as NSCLC; (2) undergoing LC thoracoscopic lobectomy in our hospital, (3) age between 50 and 85 years; and (4) with a writing agreement of informed consent. Exclusive criteria were as follows: (1) current pneumonia/asthma before the surgery; (2) received pharmacological interventions, such as antibiotics and corticosteroids, within 2 weeks; (3) with intraoperative hemorrhage greater than 1000 mL; (4) patients with conversion to thoracotomy; (5) transferred to ICU after the surgery; and (6) underwent new adjuvant radiochemotherapy. The clinical and surgical data of all included patients were prospectively analyzed.

### 2.3. Anesthesia

All the patients received general anesthesia. The protocol of induction included intravenous propofol (1–2 mg/kg), sufentanil (0.25–0.5 *μ*g/kg), and 0.2 mg/kg cisatracurium. After the induction, the patient was intubated with a double lumen endotracheal tube. During the one-lung ventilation, the ventilator was set at a tidal volume of 5–6 mL/kg, a positive end-expiratory pressure of 5 cm H_2_O, an inspiratory-to-expiratory ratio of 1 : 1.5, FiO_2_ of 100%, and a respiratory rate of 16/min.

### 2.4. Surgical Resection

Operations were undertaken by board-certified thoracic surgeons. The lung was resected using anatomical lobectomy for the patients. The lymph node was dissected using an electrocoagulation hook or ultrasonic scalpel when patients were diagnosed with LC by intraoperative frozen section [12].

### 2.5. Routine Perioperative Examinations and FeNO Measurement

Routine perioperative examinations include pulmonary function tests, and complete blood count and levels of albumin and FeNO were measured before and 24 hours after the surgery. A chest X-ray was performed 24 hours after the surgery. The spirometer (GANSHORN, Germany) was used to measure pulmonary function and record the pulmonary function parameters (such as FEV1, FEV1/FVC, DLCO etc.), whose normal values refer to the Global Lung Function Initiative (GLI) [13]. FeNO was measured using NIOX VERO (Aerocrine, CIRCASSIA). Measurement was taken step by step as the user manual instructed. The exhalation time was 10 seconds. The exhalation flow rate was 50 ± 5 mL/second. The unit of FeNO was presented in parts per billion (ppb).

### 2.6. Outcome

The primary outcome was postoperative pneumonia. Postoperative pneumonia was defined according to the criteria proposed by STS and ESTS, including new or progressive and persistent infiltrate, consolidation or cavitation found by chest radiographs, and at least one of the following must be met: fever (>38°C) without other recognized reasons; leukopenia (<4,000 WBC/mm^3^) or leukocytosis (<12,000 WBC/mm^3^); for patients >70 years old, change in mental status with purulent sputum or change in character, and respiratory secretions increasing or needing suction; onset or worsening symptoms (e.g., dyspnea, tachypnea) or clinical signs (e.g., rales, bronchial breath sounds) [14, 15]. Secondary outcomes included length of stay, drainage amount, drainage duration, and duration of antibiotic use.

### 2.7. Statistics Analysis

Data were expressed as means ± standard deviation, median (interquartile range), or the number of patients (*n*, (%)). Fisher's exact test, chi-square test, Student's *t*-test, the Kolmogorov–Smirnov test, and the Mann–Whitney *U*-test were used for comparing variables as appropriate. The Wilcoxon test was performed to compare pre-op-FeNO and post-op-FeNO in both the POP group and non-POP group. In order to investigate potential predictive factors of POP and evaluate the power of FeNO to predict POP, bivariate logistic regression analyses were performed. The receiver operating characteristic (ROC) curve was performed to evaluate the sensitivity and specificity of postoperative FeNO (post-op-FeNO) as well as the change in FeNO (∆FeNO) value for predicting the occurrence of POP in LC patients after lobectomy. All results were determined significant at a value of *P* < 0.05. Statistical analyses were conducted using SPSS software v.26.0.

## 3. Results

### 3.1. The Baseline Characteristics of the Patients

**Figure 1:**
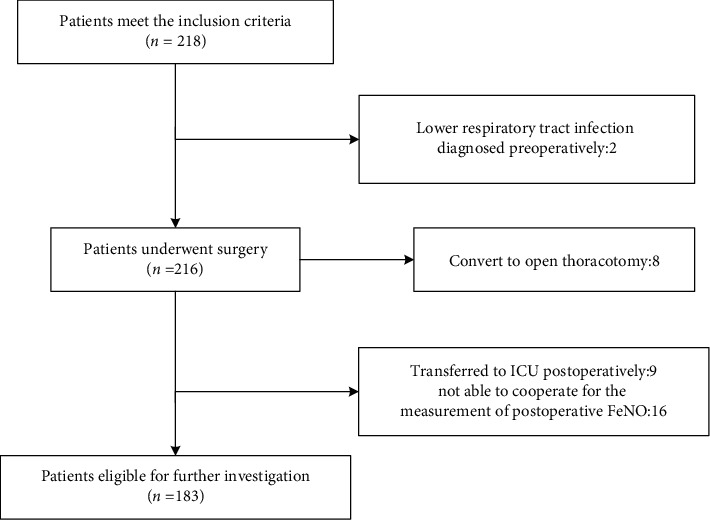
Flow chart of patient recruitment. FeNO: fractional exhaled nitric oxide; ICU: intensive care unit.

There were 218 patients enrolled in this study. 8 patients were converted to open thoracotomy. 9 patients were transferred to ICU when surgery was finished. 2 patients were diagnosed with lower respiratory tract infection preoperatively. 16 patients could not cooperate for the measurement of postoperative FeNO. Hence, finally, 183 patients were included in the study (Figure 1). The clinical characteristics of patients are shown in Table 1. Of these patients, 19 developed POP and were categorized into the POP group (Table 2), and the remaining 164 were in the non-POP group. The average age of those in the POP group was older than that in the non-POP group (61.21 ± 9.52 vs. 55.95 ± 9.75 years, *P* = 0.027). Concerning in-hospital stay, the POP group had longer total (median: 13.0 vs. 9.0 days, *P* < 0.001) and postoperative length of stay (median: 7.0 vs. 4.0 days, *P* < 0.001) than the non-POP group; and there were differences in the duration of antibiotic use (median: 5.0 vs. 2.0 days, *P* = 0.001), drainage (median: 3.0 vs. 2.0 days, *P* = 0.001), and operation time (median: 110.0 vs. 80.0 min, *P* = 0.002) between the groups, which were statistically significant.

### 3.2. Perioperative FeNO

**Figure 2:**
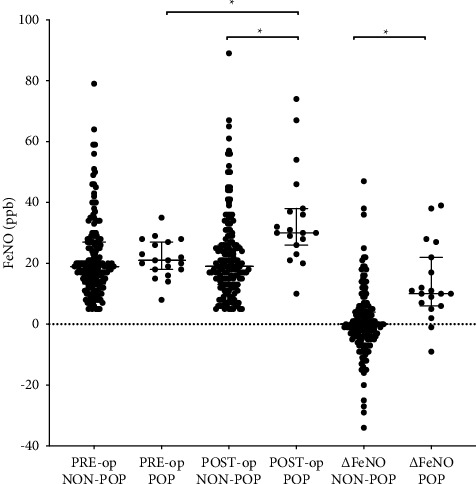
Perioperative FeNO levels and change in FeNO in the POP group (*N* = 19) and non-POP group (*N* = 164). Dot plots revealed that the post-op-FeNO and ∆FeNO were higher in the POP group than those in the non-POP group (*P* < 0.001). For patients with POP, there was a significant difference in the FeNO level before and after the surgery (*P* < 0.001). For patients without POP, pre-op-FeNO and post-op-FeNO did not show a significant difference (*P*=0.7841).

**Figure 3:**
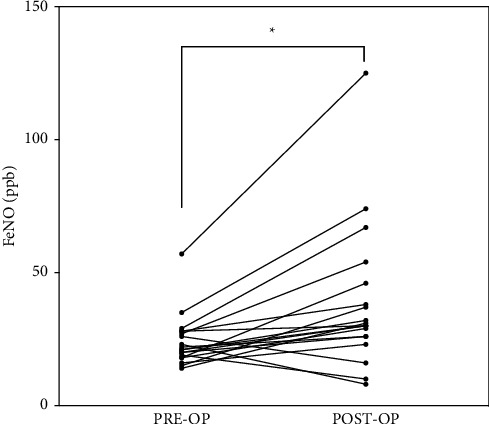
For the patients developed with POP, a trend toward increased FeNO levels was noted (*P* < 0.001). FeNO: fractional exhaled nitric oxide; PRE-OP: preoperation; POST-OP: postoperation.

In this study, changes in the FeNO concentration were observed in 170 patients, including 87 increases and 83 decreases. In the non-POP group, we did not observe a significant difference between pre-op-FeNO and post-op-FeNO (*P*=0.7841). However, for the patients with POP, post-op-FeNO was significantly higher than pre-op-FeNO (median: 30.0 vs. 21.0 ppb, *P* < 0.001). Nineteen patients developed POP, and FeNO was elevated postoperatively in 16 of them. The distribution of perioperative FeNO and ∆FeNO is shown in Figure 2. With regard to perioperative FeNO, no difference was found in preoperative FeNO between the groups (*P*=0.276); but the POP group had significantly higher post-op-FeNO (median: 30.0 vs. 19.0 ppb, *P* < 0.001) and ∆FeNO (median: 10.0 vs. 0.0 ppb, *P* < 0.001) after the surgery.  Figure 3 shows that a trend of elevated FeNO was noted in the patients who developed with POP.

### 3.3. The Optimal Cutoff of the Post-Op-NO and ∆FeNO for Predicting POP

**Figure 4:**
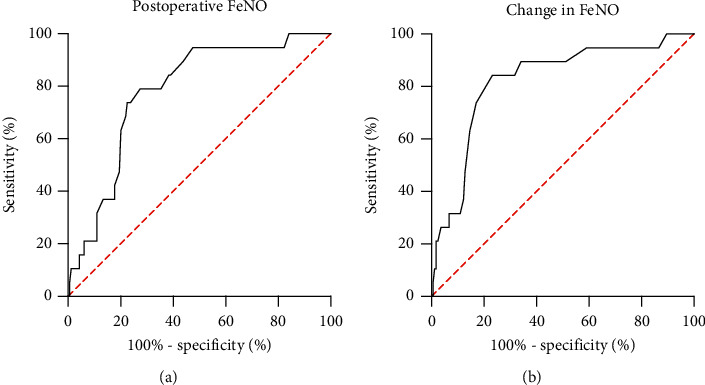
ROC curve was performed to evaluate the sensitivity and specificity of post-op-FeNO and ∆FeNO. FeNO: fractional exhaled nitric oxide; ∆FeNO: change in FeNO; ROC curve: receiver operating characteristic curve.

We selected the optimal cutoff value of the post-op-FeNO and ∆FeNO in predicting POP based on the receiver operating characteristic (ROC) curve, with the consideration of balancing the sensitivity and specificity. Therefore, we chose a cutoff value of 25 ppb for post-op-FeNO (Youden's index: 0.515, sensitivity: 78.9%, specificity: 72.6%, 95% CI: 0.713–0.838) and 4 ppb for ∆FeNO (Youden's index: 0.610, sensitivity: 84.2%, specificity: 76.8%, 95% CI: 0.757–0.873). Furthermore, a post-op-FeNO of >25 ppb indicated an 8-fold increase in odds of having POP (OR = 7.792, 95% CI: 2.692–22.553, *P* < 0.001); similarly, 7-fold for a change in FeNO of >4 ppb (OR = 7.792, 95% CI: 2.692–22.553, *P* < 0.001) (Figure 4).

### 3.4. Bivariate Analysis of FeNO and Routine Perioperative Examinations Related to POP

According to the bivariate analysis of FeNO, pulmonary function, and blood tests, three variables, including FEV1/FVC (OR = 0.948, 95% CI: 0.899–0.999, *P*=0.048), POD1 FeNO (OR = 1.048, 95% CI: 1.019–1.077, *P*=0.001), and ∆FeNO (OR = 1.087, 95% CI: 1.044–1.132, *P* < 0.001), were significantly associated with occurrence of POP (Table 3).

## 4. Discussion

This prospective study is the first to assess the FeNO distribution before and 24 hours after the VATS lobectomy to evaluate the relationship between POP and post-op-FeNO and ∆FeNO. POP, which remains a significant cause of morbidity and mortality, is one of the most common complications of patients who underwent lobectomy. For the management of these patients, post-op-FeNO and ∆FeNO seem to be noninvasive, cost-effective, and easy-to-use screening adjunct for detecting POP.

In the healthy population, the value of FeNO had a large variation. And several factors may be correlated with the value of FeNO, including age, ethnicity, and height [16–19]. Under different pathophysiologic conditions, exhaled NO might be increased or decreased, indicating inflammation within the lung and respiratory tract. On the other hand, diseases of the extra-respiratory system (e.g., diabetes and hypertension) may result in increased consumption of NO [20].

It is well known that FeNO is elevated in asthma. Besides its application in asthma, the ATS/ERS also proposed the diagnostic role of FeNO in airway infections and acute lung injury [6, 8]. For the management of perioperative patients, several studies explored the role of FeNO in patients who underwent major abdominal and cardiothoracic surgeries. Gashouta et al. [21] reported that an increase in NO concentration was correlated with acute rejection, lymphocytic bronchiolitis, or acute infection after lung transplantation. Boshier et al. [22] also found the NO concentration tended to be elevated in patients with POP who underwent esophagectomy. We have detected a significant difference in post-op-FeNO and ∆FeNO values between POP and non-POP groups. Post-op-FeNO or ∆FeNO's power to detect POP was evaluated as the ROC curve indicated. ∆FeNO seems to be a promising variable for predicting POP in VATS lobectomy. These results, as well as our study, suggest that elevated FeNO was observed in patients with POP. It was explained by studies that lower respiratory tract infection caused by bacteria would result in activation of inducible NO synthase, which subsequently leads to elevated NO production [23, 24]; but we did not observe significant changes before and after the surgery in the non-POP group. The diagnostic value of FeNO could be served as a useful adjunct in detecting postoperative pulmonary complications. Besides, of the excluded patients, two were discovered with lower respiratory tract infection and their preoperative FeNO was 68 ppb and 50 ppb, respectively. This reminds us that patients with high preoperative FeNO levels should exclude existing infection and adequate intervention should be given to them before the surgery.

A tendency toward reduction in exhaled NO postoperatively was found in 83 patients (45.4%) in this study. This trend was also noted by Jones et al.; however, their patients' size was relatively small [25]. Some studies reported that a decreased level of exhaled NO was correlated with a poor prognosis. Cuthbertson et al. found a trend toward a reduction in exhaled NO after coronary artery bypass surgery when patients developed with acute lung injury [26]. And a decrease of exhaled NO was also found in ARDS undergoing mechanical ventilation [27]. A reduction in patients who underwent lobectomy may include several reasons. First, the resected lung contributes directly to this reduction. Second, for the patients who underwent cardiothoracic surgery, ventilated associated lung injury followed by one-lung ventilation, ischemia-reperfusion-induced lung injury, and lung contusion directly caused by surgical procedures contribute to the most lung injury during operation. These kinds of lung injury may result in a reduction in FeNO hours after the operation [24, 25, 28, 29]. This can be explained by increased consumption of NO and increased diffusing capacity for NO when acute lung injury developed [30]. To determine whether a decrease in FeNO after lung resection has clinical significance, more studies are needed.

There are some limitations to this study. First, the number of POP in our study is small. We did not perform multivariate regression analysis to figure out whether post-op-FeNO and ∆FeNO are independent risk factors. But as we know, FEV1/FVC is not a reliable tool in predicting POP according to recent studies. Hence, post-op-FeNO or ∆FeNO may be a potential risk factor of POP. Second, FeNO measured after lung resection is subjected to acute lung jury caused by operation and anesthesia. Our results suggested that the operation and anesthesia did not have a significant influence on FeNO measured 24 h after the surgery. For better-predicting POP and avoiding the influence caused by operation and anesthesia, more studies are required to determine the time point for measuring FeNO. Third, post-op-FeNO was measured for the single time, which was 24 hours after the surgery. We did not measure FeNO when patients recovered from POP. And additional investigations are required to determine whether serial monitoring of FeNO could be used as an indicator to withdraw antibiotics.

## 5. Conclusion

We have observed in this prospective study that a high postoperative FeNO (>25 ppb), as well as an increased ∆FeNO (>4 ppb), may serve as a screening adjunct of detecting POP in surgical LC patients.

## Figures and Tables

**Table 1:** Baseline and clinical characteristics of the two groups.

	POP group, *N* = 19	Non-POP group, *N* = 164	*P* value
Age (years)^#^^*∗*^	61.2 ± 9.5	56.0 ± 9.8	0.027
BMI (kg/m^2^)^##^	22.7 (21.0, 25,9)	22.9 (21.3, 24.8)	0.747
Gender, (*n* (%))			0.463
Male	10 (52.6)	68 (41.5)	
Female	9 (47.4)	96 (58.5)	
Smoking status, (*n* (%))			0.168
Current smoking	8 (42.1)	41 (25.0)	
Ex-smokers or nonsmokers	11 (57.9)	123 (75.0)	
Pulmonary function			
FEV1 (L)^##^	2.40 (2.2, 2.8)	2.52 (2.2, 3.0)	0.329
FEV1/FVC^##^	79.0.(70.5–83.0)	80.5(76.2–84.3)	0.146
DLCO (mL/mmHg/min)^##^	20.5 (18.4, 24.8)	22.1 (19.2, 25.3)	0.242
Comorbidities (*n* (%))			
COPD	4 (21.1)	17 (10.4)	0.243
Diabetes mellitus	2 (10.5)	7 (4.3)	0.237
CVD	4 (21.1)	25 (15.2)	0.510
Clinical stage > II, (*n* (%))			0.538
Yes	17 (89.5)	135 (82.3)	
No	2 (10.5)	29 (17.7)	
Resection type, (*n* (%))			0.376
RUL	5 (26.3)	55 (33.5)	
RML	3 (15.8)	15 (9.1)	
RLL	6 (31.6)	28 (17.1)	
LUL	4 (21.1)	41 (25.0)	
LLL	1 (5.3)	25 (15.2)	
Blood test			
Preoperative WBC (×10^9^/L)^#^	5.9 ± 1.9	5.8 ± 1.5	0.639
Preoperative N (%)^#^	59.6 ± 16.0	57.3 ± 8.6	0.329
Preoperative EO (%)^##^	2.3 (1.8,3.7)	2.2 (1.1, 3.4)	0.516
Preoperative albumin (g/L)^#^	44.7 ± 3.4	44.4 ± 3.2	0.727
POD1 WBC^##^	9.6 (8.2, 12.0)	9.6 (7.9, 11.3)	0.453
POD1 N (%)^##^	82.0 (75.0%, 84.9)	78.4 (73.2, 82.6)	0.280
POD1 EO (%)^##^	0.4 (0.1,1.8)	0.4 (0.1, 1.4)	0.973
POD1 albumin (g/L)^#^	37.9 ± 4.0	39.1 ± 3.4	0.172
Length of stay (days)			
Total^##^^*∗*^	13.0 (10.0, 19.0)	9.0 (8.0, 10.0)	<0.001
Postoperative^##^^*∗*^	7.0 (6.0, 10.0)	4.0 (3.0, 5.0)	<0.001
FeNO value (ppb)			
Preoperative FeNO^##^	21.0 (18.0, 27.0)	19.0 (14.0, 27.0)	0.276
Postoperative FeNO^##^^*∗*^	30.0 (26.0, 38.0)	19.0 (13.0, 26.0)	<0.001
Change in FeNO^##^^*∗*^	10.0 (6.0, 22.0)	0.0 (−4.0, 4.0)	<0.001
Amount of blood loss (mL)^##^	50.0 (20.0, 200.0)	30.0 (20.0, 50.0)	0.113
Operation time (min)^##^^*∗*^	110.0 (80.0, 150.0)	80.0 (60.0, 100.0)	0.002
Duration of antibiotic use (days)^##^^*∗*^	5.0 (4.0, 6.0)	2.0 (1.0, 2.0)	0.001
Drainage duration (days)^##^^*∗*^	3.0 (3.0, 7.0)	2.0 (1.0, 3.0)	0.001

^#^Data with mean ± SD, ^##^data with median (interquartile range), and ^*∗*^data with significant differences. POP: postoperative pneumonia; BMI: body mass index; current smoking: still smoking in last 3 months; ex-smokers: quit smoking for more than 1 year; COPD: chronic obstructive pulmonary disease; CVD: cardiovascular disease including hypertension and coronary heart disease; DM: diabetes mellitus; FEV1: forced expiratory volume in 1 second; FVC: forced vital capacity; DLCO: diffusion capacity of the lung for carbon monoxide; FeNO: fractional exhaled nitric oxide; WBC: white blood cell; N: neutrophil granulocyte; EO: eosinophil granulocyte, POD: postoperative day; RUL: right upper lobe; RML: right middle lobe; RLL: right lower lobe; LUL: left upper lobe; LLL: left lower lobe.

**Table 2:** Characteristics of patients with POP.

Gender	Age (years)	Comorbidity	FEV1 (L)	DLCO (Ml/min/mmHg)	Pre-FeNO (ppb)	Post-FeNO (ppb)	Change in FeNO (ppb)	Onset of POP	Bacteria	Duration of antibiotic use (days)
F	68	COPD	1.68	16.92	23.00	28.00	5.00	POD 3	NA	5.00
M	64	COPD	1.32	19.83	26.00	36.00	10.00	POD 5	*K. pneumoniae*	7.00
F	69	DM	2.83	17.08	22.00	21.00	−1.00	POD 2	NA	5.00
M	66	—	1.89	20.85	20.00	30.00	10.00	POD 2	NA	5.00
F	53		2.40	19.72	28.00	30.00	2.00	POD 2	NA	4.00
M	56	—	3.73	35.55	20.00	26.00	6.00	POD 3	NA	6.00
M	73	COPD	2.23	16.78	35.00	74.00	39.00	POD 1	NA	4.00
F	50.	—	2.39	20.40	8.00	20.00	12.00	POD 2	NA	5.00
M	48	CVD	3.60	29.00	21.00	30.00	9.00	POD 3	NA	3.00
M	57	—	4.19	29.50	14.00	31.00	17.00	POD 2	NA	4.00
F	62	COPD	1.73	21.23	19.00	10.00	−9.00	POD 4	*P. aeruginosa*	9.00
F	62	DM	2.33	24.81	21.00	32.00	11.00	POD 2	NA	5.00
M	62	—	2.40	21.85	15.00	37.00	22.00	POD 3	NA	4.00
M	73	CVD	2.44	16.02	16.00	23.00	7.00	POD 2	*E. coli*	6.00
F	75	CVD	2.44	19.50	18.00	29.00	11.00	POD 4	NA	8.00
M	60	—	2.45	21.32	18.00	46.00	28.00	POD 3	NA	3.00
F	44	—	3.03	26.34	27.00	54.00	27.00	POD 2	NA	5.00
M	48	—	2.30	18.36	28.00	38.00	10.00	POD 4	NA	3.00
F	73	CVD	2.30	20.48	29.00	67.00	38.00	POD 2	*E. coli*	6.00

POP: postoperative pneumonia; F/M: female/male; COPD: chronic obstructive pulmonary disease; CVD: cardiovascular disease, including hypertension and coronary heart disease; DM: diabetes mellitus; FEV1: forced expiratory volume in 1 s; DLCO: diffusion capacity of the lung for carbon monoxide; POD: postoperative day; *E. coli*: *Escherichia coli*; *P. aeruginosa*: *Pseudomonas aeruginosa*; *K. pneumoniae*: *Klebsiella pneumoniae*; FeNO: fractional exhaled nitric oxide.

**Table 3:** Bivariate analysis of FeNO and routine perioperative examinations.

Variables	OR	*P*	95% CI
Preoperative FEV1	0.733	0.450	0.327–1.642
Preoperative FEV1/FVC^*∗*^	0.948	0.048	0.899–0.999
Preoperative DLCO	0.952	0.251	0.875–1.035
Preoperative FeNO	0.999	0.979	0.961–1.040
POD1 FeNO^*∗*^	1.048	0.001	1.019–1.077
∆FeNO^*∗*^	1.087	<0.001	1.044–1.132
Preoperative WBC	1.076	0.637	0.794–1.457
POD1 WBC	1.108	0.264	0.926–1.327
Preoperative EO (%)	1.067	0.554	0.856–1.329
POD1 EO (%)	0.922	0.693	0.616–1.380
Preoperative N (%)	1.027	0.322	0.974–1.083
POD1 N (%)	0.974	0.232	0.932–1.017
Preoperative albumin	1.028	0.725	0.883–1.196
POD1 albumin	0.905	0.158	0.787–1.040

OR: odd ratio; CI: confidence interval; FEV1: forced expiratory volume in 1 s; DLCO: diffusion capacity of the lung for carbon monoxide; FeNO: fractional exhaled nitric oxide; ∆FeNO: change in FeNO; WBC: white blood cell; N: neutrophil granulocyte; EO: eosinophil granulocyte, POD: postoperative day.

## Data Availability

The data used to support the findings of this study are available from the corresponding author upon request.

## References

[1] Gao S., Li N., Wang S. (2020). Lung cancer in people’s Republic of China. *Journal of Thoracic Oncology*.

[2] Deshpand R., Chandra M., Rauthan A. (2022). Evolving trends in lung cancer: epidemiology, diagnosis, and management. *Indian Journal of Cancer*.

[3] Katsura M., Kuriyama A., Takeshima T., Fukuhara S., Furukawa T. A. (2015). Preoperative inspiratory muscle training for postoperative pulmonary complications in adults undergoing cardiac and major abdominal surgery. *Cochrane Database of Systematic Reviews*.

[4] Wang D., Huang X., Wang H. (2021). Risk factors for postoperative pneumonia after cardiac surgery: a prediction model. *Journal of Thoracic Disease*.

[5] Song Y., Liu J., Lei M. (2021). An external-validated algorithm to predict postoperative pneumonia among elderly patients with lung cancer after video-assisted thoracoscopic surgery. *Frontiers in Oncology*.

[6] Dweik R. A., Boggs P. B., Erzurum S. C. (2011). An official ats clinical practice guideline: interpretation of exhaled nitric oxide levels (feno) for clinical applications. *American Journal of Respiratory and Critical Care Medicine*.

[7] Asano K., Chee C. B., Gaston B. (1994). Constitutive and inducible nitric oxide synthase gene expression, regulation, and activity in human lung epithelial cells. *Proceedings of the National Academy of Sciences of the USA*.

[8] American Thoracic Society (2005). ATS/ERS recommendations for standardized procedures for the online and offline measurement of exhaled lower respiratory nitric oxide and nasal nitric oxide, 2005. *American Journal of Respiratory and Critical Care Medicine*.

[9] ten Oever J., Mandon J., Netea M. G. (2013). Pulmonary infection, and not systemic inflammation, accounts for increased concentrations of exhaled nitric oxide in patients with septic shock. *Journal of Breath Research*.

[10] Liu D., Luo G., Luo C., Wang T., Sun G., Hei Z. (2015). Changes in the concentrations of mediators of inflammation and oxidative stress in exhaled breath condensate during liver transplantation and their relations with postoperative ARDS. *Respiratory Care*.

[11] Okamoto K., Hayashi K., Kaku R., Kawaguchi Y., Oshio Y., Hanaoka J. (2020). Impact of fractional exhaled nitric oxide on the outcomes of lung resection surgery: a prospective study. *Journal of Thoracic Disease*.

[12] Liu L., Che G., Pu Q. (2010). A new concept of endoscopic lung cancer resection: single-direction thoracoscopic lobectomy. *Surgical Oncology*.

[13] Cooper B. G., Stocks J., Hall G. L. (2017). The global lung function initiative (gli) network: bringing the world’s respiratory reference values together. *Breathe*.

[14] Seder C. W., Salati M., Kozower B. D. (2016). Variation in pulmonary resection practices between the society of thoracic surgeons and the European society of thoracic surgeons general thoracic surgery databases. *The Annals of Thoracic Surgery*.

[15] Brunelli A., Salati M., Rocco G. (2017). European risk models for morbidity (eurolung1) and mortality (eurolung2) to predict outcome following anatomic lung resections: an analysis from the European society of thoracic surgeons database. *European Journal of Cardio-Thoracic Surgery*.

[16] See K. C., Christiani D. C. (2013). Normal values and thresholds for the clinical interpretation of exhaled nitric oxide levels in the us general population: results from the national health and nutrition examination survey 2007–2010. *Chest*.

[17] Leng G., Li Z., Wang Q. (2012). Detection of exhaled nitric oxide of healthy in Nanjing. *Journal of clinical otorhinolaryngology, head, and neck surgery*.

[18] Mallol J., Aguirre V., Córdova P., Cortez E., Gallardo A., Riquelme C. (2015). Fraction of exhaled nitric oxide in healthy chilean schoolchildren aged 8–15 years. *Archaeology and Neuropathology*.

[19] Dressel H., de la Motte D., Reichert J. (2008). Exhaled nitric oxide: independent effects of atopy, smoking, respiratory tract infection, gender and height. *Respiratory Medicine*.

[20] Mummadi S. R., Hahn P. Y. (2016). Update on exhaled nitric oxide in clinical practice. *Chest*.

[21] Gashouta M. A., Merlo C. A., Pipeling M. R. (2015). Serial monitoring of exhaled nitric oxide in lung transplant recipients. *The Journal of Heart and Lung Transplantation*.

[22] Boshier P. R., Knaggs A. L., Hanna G. B., Marczin N. (2017). Perioperative changes in exhaled nitric oxide during oesophagectomy. *Journal of Breath Research*.

[23] Antus B., Csiszer E., Czebe K., Horvath I. (2005). Pulmonary infections increase exhaled nitric oxide in lung transplant recipients: a longitudinal study. *Clinical Transplantation*.

[24] Wheeler M. A., Smith S. D., García-Cardeña G., Nathan C. F., Weiss R. M., Sessa W. C. (1997). Bacterial infection induces nitric oxide synthase in human neutrophils. *Journal of Clinical Investigation*.

[25] Jones R. O., Anderson N. H., Murchison J. T. (2014). Innate immune responses after resection for lung cancer via video-assisted thoracoscopic surgery and thoracotomy. *Innovations: Technology and Techniques in Cardiothoracic and Vascular Surgery*.

[26] Cuthbertson B. H., Stott S. A., Webster N. R. (2002). Exhaled nitric oxide as a marker of lung injury in coronary artery bypass surgery. *British Journal of Anaesthesia*.

[27] Brett S. J., Evans T. W. (1998). Measurement of endogenous nitric oxide in the lungs of patients with the acute respiratory distress syndrome. *American Journal of Respiratory and Critical Care Medicine*.

[28] Marczin N. (2005). The biology of exhaled nitric oxide (no) in ischemia-reperfusion-induced lung injury: a tale of dynamism of no production and consumption. *Vascular Pharmacology*.

[29] Cui Y., Pi X., Wang C. (2015). Effects of different ventilation strategies on exhaled nitric oxide in geriatric abdominal surgery. *Journal of Breath Research*.

[30] Lakshminrusimha S., Suresh M. V., Knight P. R. (2013). Role of pulmonary artery reactivity and nitric oxide in injury and inflammation following lung contusion. *Shock*.

